# Patient counselling on opioids by pharmacy technicians: A mixed-method study to explore facilitators and barriers

**DOI:** 10.1016/j.pecinn.2025.100382

**Published:** 2025-02-17

**Authors:** Elsemiek A.W. Jansen-Groot Koerkamp, Irem Simsek, Eman Badawy, Mette Heringa, Marcel L. Bouvy

**Affiliations:** a SIR Institute for Pharmacy Practice and Policy, Theda Mansholtstraat 5B, 2331 JE Leiden, the Netherlands; b Division of Pharmacoepidemiology and Clinical Pharmacology, Utrecht Institute for Pharmaceutical Sciences, Utrecht University, Universiteitsweg 99, 3584 CG Utrecht, the Netherlands

**Keywords:** Pharmacy technicians, Counselling, Primary health care, Opioids, Opioid-related disorder

## Abstract

**Objectives:**

This study investigates community pharmacy technicians' (PTs) counselling practices for patients prescribed opioids and identifies facilitators and barriers influencing their counselling behaviour.

**Methods:**

A sequential exploratory mixed-method study involving interviews and a questionnaire was conducted among PTs, working in Dutch community pharmacies. PTs were recruited via professional networks, panels and social media. Inductive thematic analysis was performed for interviews. Descriptive statistics of questionnaires was performed and associations between behaviour of discussing dependency and background characteristics (1), barriers (2) and beliefs (3) were tested.

**Results:**

Nineteen topics emerged from 18 interviews. Out of 252 questionnaire-respondents, most PTs consistently discussed dosage and common side effects during the first opioid dispense. Although 92 % considered discussing opioid dependency important, only 62 % frequently performed it. Barriers included a lack of information about the indication (*p* = 0.012), lack of work agreements (*p* = 0.017), time (*p* = 0.022), feeling insecure (*p* = 0.041), less work experience (*p* = 0.025) and the belief that prescribers are responsible for discussing opioid dependency with patients (*p* = 0.018).

**Conclusion:**

The high importance that PTs place on counselling patients on opioid dependency does not match their behaviour. To close this gap and optimize the role of PTs in promoting safe opioid use, organizational and competency-related barriers must be addressed. This includes clear work protocols, communication training and including the indication on opioid prescriptions.

**Innovation:**

This research focuses on an underexplored group involved in patients' opioid management, crucial for designing effective interventions, as PTs frequently have direct patient contact.

## Highlights

• This study explores the role of an underexplored group involved in opioid management.
• The role of pharmacy technicians in promoting safe and effective opioid use can be improved.
• Including indication on an opioid prescription will benefit appropriate opioid counselling by pharmacy technicians.
• Pharmacy technicians require training and protocols to adequately discuss the risks and benefits of opioids.

## 1 Introduction

Opioid use significantly increased over the years, mainly in the United States [1]. In Europe, the use of opioids has risen too [[2], [3], [4], [5]]. This increase is mainly caused by opioid use as a treatment for chronic non-cancer pain. [2,6]. However, the long-term effectiveness of opioids in chronic non-cancer pain is debatable [[7], [8], [9]]. Moreover, opioid use is associated with multiple risks such as sedation, constipation and respiratory depression, as well as an increased risk of myocardial infarction, falls, fractures and all-cause mortality [[10], [11], [12]].

Health care providers play a crucial role in educating patients about opioid safety and informing patients about the risks of opioid use. In case of cancer-related pain or pain at end-of-life prioritizing quality of life, including adequate pain management is paramount [13] and opioid dependency should not be feared. In case of opioid treatment for chronic non-cancer pain, opioid dependency should be considered as a serious risk and thereby evaluating the risk-benefit balance. Studies have shown that patients on long term opioid treatment often receive insufficient and inadequate information about opioids, with a longing for comprehensive education on opioid risks [[14], [15], [16]]. Patients on long term opioid therapy want more frequent evaluations of pain management and opioid efficacy by both general practitioners (GP) and community pharmacists (CPs) [14].

In the Netherlands, health care providers involved in opioid therapy are responsible for providing information and conducting treatment evaluations. Community pharmacies serve as the primary setting for medication dispensing and counselling. Dutch pharmacists are by law co-practitioners, sharing responsibility for pharmacotherapy with physicians. Dutch pharmacy technicians (PTs) work independently under the supervision and responsibility of the pharmacist. PTs have the most extensive patient contact within the pharmacy and play a crucial role in patient consultations [17]. PTs verify and dispense incoming prescriptions. During a first dispense, PTs inform patients about the intended benefits of new medications, dosing regimen, duration of action, potential side effects, and interactions. They are supported by a pharmacy information system to provide this information. PTs also assist in follow-up consultations to monitor patient progress and address any issues, which enhances patient understanding and adherence to treatment. To fulfil these responsibilities, PTs must complete a three-year vocational education program and they are stimulated to engage in ongoing professional development to stay current in their field.

However, there is limited understanding of how PTs counsel patients with opioid prescriptions. Studies from the United States highlight challenges faced by PTs, such as feeling under-resourced and ill-quipped to adequately serve opioid users, along with the presence of stigma associated with patients' opioid therapy [18,19].

This study aims to investigate how pharmacy technicians in the Netherlands counsel patients who are prescribed opioids, as well as which facilitators and barriers influence their counselling behaviour.

## 2 Methods

### 2.1 Study design

This research is a mixed-methods study, used in an exploratory sequential design [20,21]. First, a qualitative approach (interviews) was used to identify topics relevant for opioid counselling practices by PTs (step 1). Second, a quantitative approach (questionnaire) was used to quantify the relevance of the identified topics (step 2).

### 2.2 Step 1 interviews

#### 2.2.1 Interview guide

A semi-structured interview guide was drafted by IS (pharmacists-in training) and MH (pharmacist). MH had a training in qualitative research. The interview guide focussed on topics about the information provision during consultations with patients about opioids, considerations of PTs to talk or not to talk about specific subjects, and opinions of PTs on chronic opioid use by patients. The interview guide with 28 questions was discussed within the research team till a final version was compiled. It was pilot tested with the first interview and refined as needed (supplementary file 1).

#### 2.2.2 Recruitment and data collection

Eligibility criteria included being a Dutch-speaking pharmacy technician working in a Dutch community pharmacy. Participants for the interviews were recruited through pharmacies affiliated with the Utrecht Pharmacy Practice network for Education and Research [22]. A variety of pharmacies was approached in both urban and rural settings, in healthcare centres and stand-alone, large and small pharmacies. Data saturation was defined as the point at which no new main codes emerged [23]. Parallel analysis was conducted alongside the interviews. The interviews were conducted online through MS Teams, by telephone or in-person. Oral informed consent was obtained from all study participants before the start of the interview. All interviews were audio-recorded.

#### 2.2.3 Interview analysis

Interview audio-recordings were transcribed verbatim (IS). All audio recordings were deleted after analysis of the data. Transcripts were read repeatedly for familiarization with the data. First, two interviews were double coded (IS and EB). Thematic analysis involving an inductive approach was used to identify themes and topics. Coding discrepancies were discussed with a third researcher (MH). One researcher (IS) conducted the initial categorisation of the codes. Concept themes and topics were evaluated with the research team. NVivo (version 12, QRS International) was used for data management and content analysis.

### 2.3 Step 2: questionnaire

#### 2.3.1 Questionnaire design

The topics emerged from the interviews were used to develop a questionnaire. The questionnaire was designed to examine the relevance of the identified topics among more PTs. The first section of the questionnaire consisted of background characteristics, including current position, age, gender, work experience and pharmacy setting. The second section focused on the current opioid counselling behaviour. The third section focused on actions performed in case of concerns about a patient's opioid use. The fourth section addressed participant's considerations on providing information about opioid dependence, while the last section focused on PTs' beliefs about opioid use and the need for further education. The majority of the questions where on a five-point Likert scale (strongly disagree, disagree, neutral, agree, strongly agree; or: never, rarely, sometimes, often, always). The following definitions were used: opioids in this questionnaire refers to strong opioids such as oxycodone, fentanyl, and morphine. Less potent agents such as tramadol and codeine are not considered here. Long-term use of opioids is defined as continuous use for longer than three months.

Five PTs pre-tested the questionnaire for clarity, feasibility, and duration. Based on their feedback it was revised to a final version of 53 questions (approximately 10 min) (Supplementary file 2).

#### 2.3.2 Recruitment and data collection

Pharmacy technicians working in a community pharmacy in the Netherlands were invited to complete the online questionnaire. All respondents were voluntary and anonymous. The questionnaire was distributed through the Panel on practical research for Pharmacy Employees (PAM), and social media. The PAM-panel consists of circa 1000 pharmacy employees. About 90 % of the PAM panel are PTs, the remaining 10 % are other pharmacy staff members such as pharmacy consultants (specialized PTs) or pharmacy managers (mostly also PTs). Recent panel consultations indicate that the response rate for online questionnaires in the PAM panel is around 12 % [24,25]. To recruit more participants, we utilized Linked-In and Facebook as outreach platform. Free posts were shared in Dutch PT groups, by researchers and related business-pages. To increase validity of participants working in a community pharmacy, their profession was the first question after informed consent. Data was collected in March and April 2022.

#### 2.3.3 Data analysis

Completed questionnaires were included in the analysis. IBM SPSS version 28.0 was used to prepare the data and complete the analysis. Specialized PTs and pharmaceutical managers were also included in the pharmacy technician sample, as most of them are also involved in patient counselling and opioid dispensing. Associations between informing patients about dependency during first dispense (behaviour) and background characteristics, barriers and beliefs were analysed using chi-square analysis (*p* < 0.05). Given the scope of the questionnaire, informing patients about dependency during first dispense was selected as primary behaviour item. To increase robustness, both five-point Likert scales were reduced. The frequency scale used for responses was merged into three categories: ‘never-rarely’, ‘sometimes’, and ‘often-always’. The other frequency scale for barriers and beliefs was merged into two categories: ‘(strongly-)disagree-neutral’ and ‘(strongly-)agree’.

## 3 Results

### 3.1 Step 1: interviews

A total of 18 PTs, all female, were interviewed. Interviews lasted approximately 15 min. Data saturation occurred after 15 interviews. However, with three more interviews already scheduled, it was decided to proceed with all three. Table S1 shows the interviewed participants' demographics (supplementary file 3).

Twenty topics emerged from the interviews, merged to four themes: dispensing process (theme 1–7 topics), counselling process (theme 2–2 topics), reasons for (not) discussing risk of dependency (theme 3–3 topics) and beliefs of PTs about opioids (theme 4–7 topics) (Table 1). Interviews revealed that dispensing or discussing laxatives is common, mainly because it is mandated by pharmacy protocols (theme 1). However, based on the second theme, PTs did not always counsel patients on risks of chronic opioid use and addiction. Given reasons (theme 3) were that it is dependent on the indication, patients not being open for information, concerns about alarming patients and provoking negative reactions. In addition, many agreed on the primary responsibility of the prescriber for providing risk information (theme 3). PTs beliefs that patients on long-term opioid therapy often receive insufficient guidance from health care providers. Opinions varied on whether counselling protocols should be adapted to address these issues. In addition, PTs highlighted the difficulty of distinguishing whether a patient is addicted and patients who actual require opioids for pain management (theme 4).

**Table 1:** Themes and topics emerged from interviews with PTs.

Theme	Topics	Selected quotes from interview
Dispensing process	actions in case of chronic opioid use	‘I can't remember us taking any action from the pharmacy for a user of more than three months. And to be honest, it wouldn't even stand out to us if we look in the system because we don't receive any alerts or anything like that for a user of more than three months. I wouldn't really think about it on my own.’(PT 12) ‘If people use it continuously, then we consult the doctor about why someone is using it for such a long time.’ (PT4)
	actions in case of early refill	“The system also gives an alert if someone is repeating too early. Then you can look into it. Often, you just put it side by side, like ‘hey, that's two weeks earlier.’ How is someone using it? Is the prescription too early? Put a question mark next to it and ask about the usage at the counter. To check if someone might be taking more tablets.” (PT1)
	co-dispensing laxatives	‘And then, of course, the constipation is the most important, which is why we standardly give out macrogol sachets.’ (PT17)
	source of information for first dispense	‘Well, actually, we go through the VI leaflet (provided by pharmacy information system), you know. We just have VI leaflets, and we simply go through them.’ (PT16)
	source of information for first refill	‘Also pharmacy information system leaflets or notes.’ (PT15)
	duration first dispense	‘Yes, exactly, that's actually the case with all medications, that for the first dispensation, it is given for 2 weeks. Also, of course, with opioids.’ (PT3) ‘We dispense for a maximum of one month’ (PT18)
	regular dispensing checks	‘When I receive a one-year prescription, alarm bells definitely go off for me, wondering if this is really the intention. Then I contact the doctor because I want to know what's going on. Often, it's not the intention, and it's often not feasible either.’ (PT9)
Counselling process	guiding by pharmacy information system	‘That they (patients) can become dizzy, feel nauseous, their reaction time can be affected, (…), they can also start to hallucinate, but we don't always mention that. Sometimes, they find it a bit frightening.’ (PT5) ‘During the first dispensation, they always receive a personalized first dispensation letter. After that, they are always welcome if they need additional information from the pharmacy.’(PT5)
	risks of chronic use	‘I don't actually say anything about it being potentially addictive.’(PT8)
Reasons for (not) discussing risk of dependency	depends on patient	‘Sometimes you have people who suddenly burst into tears because they've just been told that they are out of treatment (palliative). So, I focus very much on what the person is open to, what they need at that moment.’ (PT18)
	responsibility prescriber	“I'm not going to scare people by saying, ‘Oh, this might be addictive, you need to stop.’ I really think that's something for the general practitioner.” (PT1)
	lack of information in pharmacy information system	‘No, there's no reason for that, it's just, yes, that's actually the case with all medications—that when you dispense, you just go over the most important points.’(PT16)
Beliefs of PTs about opioids	opioid use by PTs themselves	‘Yes, so I would use it very briefly.’(PT18)
	prescriptions are prescribed too easily	‘I sometimes find it too easily prescribed. That's my opinion, actually.’(PT4)
	insufficient guidance by HCP	‘No, I think it's due to the tolerance of the medication. After a while, it's no longer effective. Because they think they have the best medication they can take. I speak from experience with people who use this quite a lot. They think there's nothing more after that medication. So, they believe this is the worst, and that there's nothing else they can do. And there's actually too little support provided in this area. And I think that's the problem with opioid use.’ (PT9)
	education demands	‘Specifically about opioids, I don't think so, because I think everyone pretty much knows that. But if you had tips in a course about how to prevent such an addiction, with education, then I think it would be useful for everyone.’(PT17)
	dependency underexposed in patient information	‘In my opinion, the negative effects, such as tolerance and side effects, should be discussed more. So that people know what they are getting into and understand that it might be really short-term and can lead to dependency very quickly. It is mentioned, but I think more emphasis should be placed on it.’(PT6)
	inappropriate chronic opioid use	‘No, I do think that people who use it chronically handle it carefully.’ (PT3) ‘Yes, I think so. For us, it is very difficult to determine when someone crosses the line and actually no longer needs it, and therefore should start tapering off. We don't really get to see or hear that. You don't have that kind of good conversation with your patients. (…)’ (PT17)
	duration of opioid treatment	‘If the patient needs it, and they are in real pain and it alleviates the pain, then they should continue using it. It can be very, yes, it's really dependent on the person, I think.’(PT16)

### 3.2 Step 2: questionnaire

The questionnaire was completed by 252 respondents (138 through PAM panel (response rate 138/1102 = 12.5 %) and 114 through social media). Although the distribution across Dutch provinces did not perfectly align with the number of employed assistants per province, it was reasonably proportional, and all provinces were represented (3 to 65 PTs per province, median 13.5).

Aligned with national data [26], 98 % of respondents were female (see Table 2). According expectations, approximately 90 % were PTs (non-specialized), with the remaining 10 % consisting of pharmacy staff members, such as pharmacy consultants (specialized PTs) or pharmaceutical managers (often background as PT). The majority of all respondents (further: PTs) had >30 years of work experience (38.2 %). The study included participants from across the Netherlands. Compared to the PAM panel, the social media group was younger, with almost 20 % aged 18–30 year (this age group that was not represented in the PAM panel), while the PAM panel had a higher proportion of older individuals with more experience.

**Table 2:** Characteristics of questionnaire respondents.

	All respondents (n = 252)	PAM-panel (*n* = 138)	Social media (*n* = 114)
Female	247 (98.0 %)	137 (99.3 %)	110 (96.5 %)
Pharmacy technicians (non-specialized)	226 (89.7 %)	121 (87.7 %)	105 (92.1 %)
Pharmacy consultants (specialized PTs)	23 (9.1 %)	14 (10.1 %)	9 (7.9 %)
Pharmaceutical managers	3 (1.2 %)	3 (2.2 %)	0 (0 %)
Mean age (in years ±SD)	48.6 (±11.3)	52.0 (±9)	44.4 (±12.5)
Age groups:			
18–30 years	22 (8.7 %)	0 (0.0 %)	22 (19.3 %)
31–45 years	69 (27.4 %)	39 (28.3 %)	30 (26.3 %)
46–60 years	124 (49.2 %)	70 (50.7 %)	54 (47.4 %)
>60 years	37 (14.7 %)	29 (21.1 %)	8 (7.0 %)
Mean years in practice⁎ (±SD)	26.1 ± 11.7	29.7 (±9.1)	21.5 (±12.8)⁎
Groups:			
0-10 years	34 (13.5 %)	1 (0.7 %)	33 (29.2 %)
11–20 years	44 (17.5 %)	25 (18.1 %)	19 (16.8 %)
21–30 years	77 (30.6 %)	46 (33.3 %)	31 (27.4 %)
>30 years	96 (38.2 %)	66 (47.8 %)	30 (26.5 %)
Location of pharmacy			
in a healthcare center	124 (49.2 %)	59 (42.8 %)	65 (57 %)
not in a healthcare center	127 (50.4 %)	79 (57.2 %)	48 (42 %)
unknown	1 (0.4 %)	0 (0.0 %)	1 (0.9 %)

PT = pharmacy technician, SD = standard deviation.

⁎ *one missing value.*

**Fig. 1:**
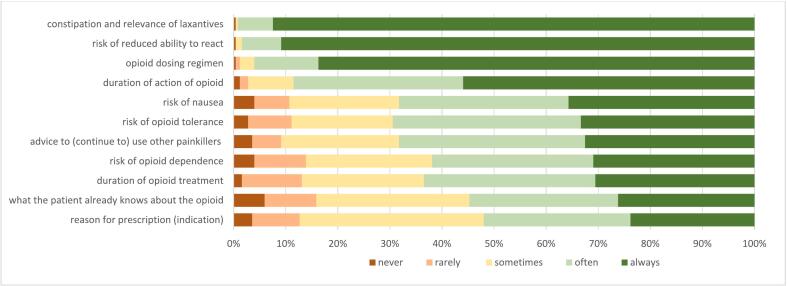
Provided information at first dispense of an opioid (*n* = 252) (% of respondents) Data is shown in supplementary file 4.

At first dispense of an opioid, the majority of the respondents always discuss the risk of constipation (and the importance of using laxatives) (92.5 %), the risk of reduced responsiveness (90.9 %) and the dosing regimen (83.7 %) (Fig. 1). Furthermore, more than half of the participants always or often provide information on the duration of the effect of opioids (88.5 %), risk of tolerance (69.4 %), the risk of nausea (68.2 %), advice about (continued) use of non-opioid painkillers (68.2 %), treatment duration (63.5 %) and risk of opioid dependency (62.0 %). However, the indication of the opioid is less frequently discussed (52.0 % often or always). PTs with more than 10 years of experience are more likely to discuss dependency (*p* = 0.025) compared to those with less experience (See Table S6, Supplementary file 4).

**Fig. 2:**
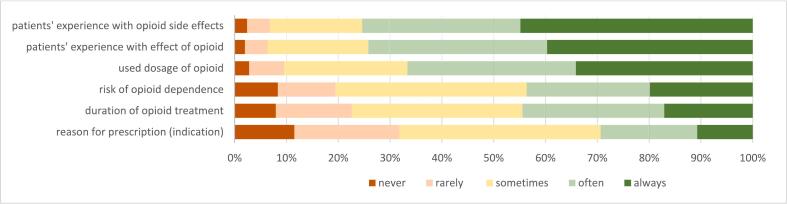
Provided information at first refill of an opioid (*n* = 252) (% of respondents) Data is shown in supplementary file 4.

In case of a first opioid refill, the majority of respondents address the patient's experience with the opioid's effects (74.2 % often and always) and side effects (75.4 %) and the used dosage of the opioid (66.6 %) (Fig. 2). Fewer respondents provide information about the duration of the opioid treatment (44.5 %) and the indication (29.4 %). During this refill, 19.8 % of the respondents always discuss opioid dependence, and 23.8 % discuss it frequently.

When dispensing an opioid, a minority of respondents reported that they always knew if the opioid is prescribed for cancer or non-cancer pain (6.3 %), while a more PTs reported knowing it often (32.9 %) or sometimes (41.7 %). Nearly one-fifth of the PTs (19.1 %) reported never or rarely knowing whether the opioid was prescribed for cancer of non-cancer pain. Similarly, only a minority always differentiates the information they provide about opioids between cancer and non-cancer pain patients (11.9 %), with more than half of the respondents (57.9 %) making this distinction often (35.7 %) or sometimes 22.2 %). Thirty percent (30.2 %) never or rarely differentiate their counselling practice based on whether the patient has a cancer diagnosis or not.

Almost all PTs have concerns about their patients' opioid use, when patients frequently request a refill prescription before they should have run out of the previous prescription (95.7 % (strongly) agree) or when patients regularly visit the pharmacy without a prescription (91.2 % (strongly) agree). Additionally, concerns arise when the dosage of the opioid has been increased several times (76.2 % (strongly) agree) or when patients use opioids for more than three months (65.5 % (strongly) agree).

When such concerns arise, most PTs consult the pharmacist (78.2 % often and always) or discuss opioid use with the patient (67.1 %). PTs also utilize the option to contact the prescriber (60.7 %). A minority make agreements with the patient regarding refill interval (27.8 %). When PT have concerns about patients' opioid use two-thirds of PTs never (34.5 %) or rarely (35.7 %) dispense the opioid without taking extra action, while a smaller proportion sometimes (22.6 %), often (6.3 %) or always (0.8 %) do.

All respondents suspected to have opioid dependent patients in their pharmacy. In the Netherlands, a pharmacy has an average of 8200 patients [27]. One third of respondents (34.1 %) estimated to have more than ten opioid dependent patients in their pharmacy, a quarter (26.2 %) estimated to have 6 to 10 opioid dependent patients. Another quarter (25.4 %) estimated to have one to five opioid dependent patients in their pharmacy and 14.3 % of PTs could not make an estimation.

**Fig. 3:**
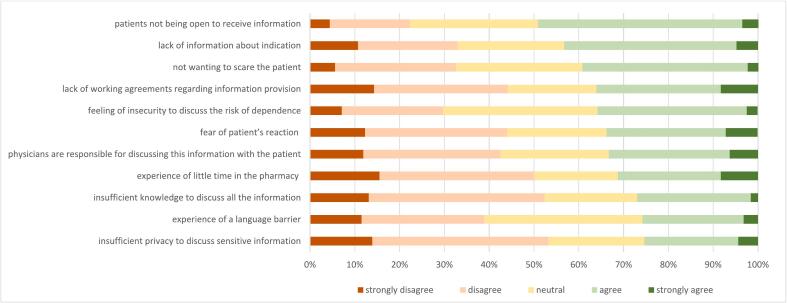
Potential barriers to discussing the risk of opioid dependence in long-term opioid use (n = 252) (% of respondents) Data is shown in supplementary file 4.

Potential barriers to discussing the risk of opioid dependence are presented in Fig. 3. Patients' reluctance to receive information was identified as the major barrier to providing information about the risk of dependence by approximately half of the technicians (49.2 %). A third (33.7 %) experienced fear of the patients' reaction and 39.3 % did not want to scare the patient about the risk of dependence. More than 40 % of the PTs (43.3 % (strongly) agree) indicate that not knowing the indication is a barrier to discussing the risk of opioid dependency, and those who agree to this barrier are less likely to discuss dependency during first dispense.(*p* = 0.012) (See Table 3).

**Table 3:** Associations between potential barriers for discussing the risk of opioid dependence and dispensing behaviour.

Barriers to discussing the risk of opioid dependence		During first dispense PT discuss the risk of opioid dependence			
		never-rarely	sometimes	often-always	*p*-value
insufficient privacy to discuss sensitive information	(strongly) disagree-neutral	24 (68.6 %)	47 (77.0 %)	117 (75.0 %)	0.645
	(strongly)-agree	11 (31.4 %)	14 (23.0 %)	39 (25.0 %)	
experience of the language barrier	(strongly) disagree-neutral	26 (74.3 %)	45 (73.8 %)	116 (74.4 %)	0.996
	(strongly)-agree	9 (25.7 %)	16 (26.2 %)	40 (25.6 %)	
insufficient knowledge to discuss all the information	(strongly) disagree-neutral	21 (60.0 %)	47 (77.0 %)	116 (74.4 %)	0.161
	(strongly)-agree	14 (40.0 %)	14 (23.0 %)	40 (25.6 %)	
experience of little time in the pharmacy	(strongly) disagree-neutral	20 (57.1 %)	50 (82.0 %)	103 (66.0 %)	**0.022**
	(strongly)-agree	15 (42.9 %)	11 (18.0 %)	53 (34.0 %)	
physicians are responsible to discuss this information with the patient	(strongly) disagree-neutral	16 (45.7 %)	43 (70.5 %)	109 (69.9 %)	**0.018**
	(strongly)-agree	19 (54.3 %)	18 (29.5 %)	47 (30.1 %)	
fear of patient's reaction	(strongly) disagree-neutral	19 (54.3 %)	47 (77.0 %)	101 (64.7 %)	0.061
	(strongly)-agree	16 (45.7 %)	14 (23.0 %)	55 (35.5 %)	
feeling of insecurity to discuss the risk of dependence	(strongly) disagree-neutral	19 (54.3 %)	47 (77.0 %)	96 (61.5 %)	**0.041**
	(strongly)-agree	16 (45.7 %)	14 (23.0 %)	60 (38.5 %)	
lack of working agreements regarding information provision	(strongly) disagree-neutral	15 (42.9 %)	39 (63.9 %)	107 (68.6 %)	**0.017**
	(strongly)-agree	20 (57.1 %)	22 (36.1 %)	49 (31.4 %)	
not wanting to scare the patient	(strongly) disagree-neutral	16 (45.7 %)	41 (67.2 %)	96 (61.5 %)	0.109
	(strongly)-agree	19 (54.3 %)	20 (32.8 %)	60 (38.5 %)	
lack of information about indication	(strongly) disagree-neutral	12 (34.3 %)	39 (63.9 %)	92 (59.0 %)	**0.012**
	(strongly)-agree	23 (65.7 %)	22 (36.1 %)	64 (41.0 %)	
patients not being open to receive information	(strongly) disagree-neutral	16 (45.7 %)	37 (60.7 %)	75 (48.1 %)	0.202
	(strongly)-agree	19 (54.3 %)	24 (39.3 %)	81 (51.9 %)	

Note: Statistical significance of differences tested by Chi-square test; Bold refers to p < 0.05 (significant).

Another factor influencing whether the risk of dependency is discussed included the lack of working agreements in the pharmacy regarding information provision about opioids (*p* = 0.017). In addition, PTs were less likely to discuss opioid dependency when they feel insecure (*p* = 0.041), experienced limited time (*p* = 0.022) or had the opinion that the prescriber is responsible to discuss this information with the patient (*p* = 0.018).

**Fig. 4:**
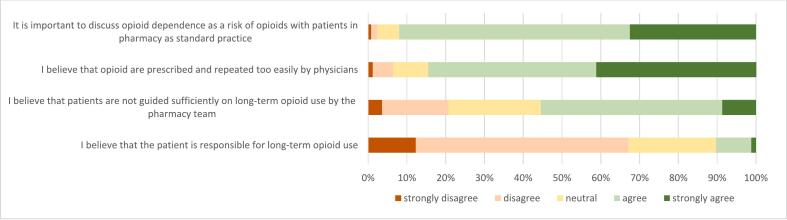
Beliefs of pharmacy technicians on long-term use of opioids (n = 252) (% of respondents) Data is shown in supplementary file 4.

Two-thirds of PTs disagreed with the idea that patients themselves are responsible for long-term opioid use (67.1 %) and more than half felt that patients are not adequately guided by the pharmacy team (55.5 %). The majority expressed concerns about the ease with which patients receive opioid prescriptions (84.6 %) and supported the notion that discussing opioid dependence as a risk should be a standard part of care (92 %) (Fig. 4). Those beliefs were not statistically significant related to informing patient about dependency at first dispense (Table S7, supplementary file 4).

While a majority of PTs disagrees with the statement that insufficient knowledge prevents discussing relevant information including dependence (52.4 %), many PTs experience a need for education on opioid dependence and its prevention (58.7 %). Additionally, 46.0 % expressed a need to gain extra knowledge about pain treatment and alternatives for opioids. Training in recognizing patients with (risk of) addiction (44.8 %) and communication skills, including motivational interviewing to encourage and support patients during tapering (43.3 %), were also identified as areas for further development.

## 4 Discussion and conclusion

### 4.1 Discussion

This study explored the information provided by Dutch PTs during opioid dispensing, as well as the facilitators and barriers they encounter. During the first opioid dispensing, PTs consistently covered essential information such as dosage regimen, the risk of impaired responsiveness, and the risk of constipation, including the importance of using laxatives. However, topics such as the indication for prescribing, the duration of treatment, and advice on alternative painkillers were less frequently addressed. Notably, although more than 90 % of the PTs emphasized the importance of discussing the risk of opioid dependence, this was often not discussed. PTs encountered various barriers that hindered them from discussing opioid dependence, including patient reluctance to receive information, concerns about alarming patients, and a lack of available indication information. Facilitators include training in communication skills, and protocols within the pharmacy on opioid counselling.

PTs believe that prescribers easily prescribe and refill opioids, and that the pharmacy team does not adequately guide patients on long-term opioid therapy. Only one third of participants always mention the risk of dependency at a first prescription. Previously, we observed that approximately half of community pharmacists (55.7 %) report that they inform patients about the risk of dependence at the first dispense, and 38.2 % do so when refilling prescriptions [28]. These self-reported counselling practices by pharmacists align reasonably with the practices reported by technicians in this study.

Worldwide, there is limited understanding of how PTs counsel patients with opioid prescriptions. To our knowledge, no other European studies have investigated the role of PTs in opioid counselling. Dependent on country and region, responsibilities of pharmacists and PTs in (opioid) dispensing and counselling differs [29]. PTs in the Netherlands completed a 3-year vocational education program. Pharmacy technicians dispense medication, informing patients about their medications and controlling the stock of the pharmacy. Guided by work protocols they check every prescription for dosage, form of the medication, duration of treatment, interactions and contraindications. They provide the patient information about the medicine, mainly at first dispense [30]. Research showed that Dutch PTs adequate counsel patients with dermatitis and give instructions how to use inhalation devices [31,32].

Compared to other European Union countries, the Netherlands have the lowest number of pharmacists per 100.000 inhabitants, namely 22 [33,34]. Dutch pharmacists completed six-year academic education and a masters' degree. Since there a relative low number of pharmacists, PTs have a sizeable role in the pharmacy. Like in the Netherlands, PTs in the US are also often the first and most frequently pharmacy staff to interact with patients with opioid prescriptions [19]. In the US, the number of pharmacists per 100.000 inhabitants is about 96 [33]. US studies reports challenges such as facing stigma, feeling under-resourced and ill-quipped to adequately serve opioid users [18,19]. Safety counselling among American pharmacists is generally limited to informing patients about potential side effects of opioids [35]. Barriers such as lack of training, time, self-efficacy in communication have been reported as reasons for limited counselling and difficulty discussing the risks of opioids with patients [35]. Those barriers were also reported by about one-third of the Dutch PTs. However, in this study, more PTs experienced barriers as patient's reluctance to receive information, concerns about alarming patients, and insufficient information of indications of opioids. This implies that Dutch PTs might be more mindful of avoiding anxiety in patients. This could be linked to the absence of an opioid crisis in the Netherlands on the scale seen in the US.

Consistent with the experiences of PTs, Dutch pharmacists have also reported to experience lack of training in prevention of long-term use and tapering schedules [28]. Pharmacists partially train their PTs themselves and serve as their backup. When the pharmacist himself lacks capabilities, does not create the right opportunities and conditions, or lacks motivation, he is unable to effectively support their PTs. This study showed that PTs were significantly less likely to bring up opioid dependency during the first opioid dispensing if they were pressed for time, lacked information regarding the indication or work agreements, or felt insecure to discuss the risk of dependency with the patient. Improvement in the capabilities of community pharmacists and PTs, and creating opportunities including sufficient time, information, confidence in counselling practices, privacy and protocols, potentially give rise to increased community pharmacists' and PTs involvement in opioid misuse prevention services [36].

The frontline presence of PTs enables them to engage with patients at different moments, offering opportunities for timely intervention to prevent inappropriate long-term use. For instance, the duration of opioid treatment is always discussed during first dispense by 31 % of PTs and around 17 % during the first refill. This percentage may decline further as the number of refills increases. Despite the opportunity to interact with patients at different moments, patients lack risk education during the initiation of treatment, medication evaluations and initiation of tapering conversations [14]. An observational study in four pharmacies showed that at repeat prescriptions of several medicines, PTs never discuss observed (side) effects, seldom discuss adherence, and that PTs did not encourage patient participation and rarely explore their needs or concerns. Patients also never initiated a discussion about adherence or experienced effects [30]. Davies et al. (2024) showed that about two-third of patients with non-cancer pain on long-term opioid therapy experienced no discussion about their use, side effects or potential dependency during opioid refills. In addition, most patients mentioned their pharmacy team had rarely commented on their prolonged use of opioids and mostly only in case of potential overuse. Only one of 25 patients mentioned receiving comments that made him re-evaluate his long-term opioid use, which he appreciated about the pharmacy [14]. It appears that patients' experiences are more negative than what PTs reported about their counselling practices. Several possible explanations include: 1) PTs provided socially desirable answers and practice inferiorly to what they claim, 2) patients in the study of Davies et al. often had been using opioids for many years possibly starting at a time when there was less emphasis on the risks of opioids, 3) patients may have forgotten the information provided to them [37]. These factors highlight the need for improvement in counselling practices and regular evaluations to ensure that important information about opioid risks is effectively communicated and retained.

Emphasizing the importance of understanding patients' indications for opioids to tailor counselling effectively, this study advocates for writing the indication on the opioid prescription by the prescriber. During opioid dispenses, only a third of PTs often or always knew the indication of the opioid. A part of the PTs probably lacks the capability to ask the patient about the indication or do not want to scare the patient by asking the indication. The Dutch law obliges prescribers to record the indication on the prescription for 23 medicines, none of them being opioids or other pain killers [38]. Research showed that most patients have no objection with mentioning the indication on prescription, it benefits patient safety and enables pharmacists to work more effective [39,40]. In the management of cancer-related pain or pain at end-of-life the focus should not be on risks like opioid dependency. However, in the context of non-cancer pain, these risks should be addressed to ensure a careful evaluation of the risk-benefit balance. Less than half of PTs distinguished their information provision based on the indication (cancer and non-cancer-related pain). Being unfamiliar with the indication was a barrier to discuss opioid dependency, as was the opinion that the prescriber is responsible for discussing this information with the patient. These findings underscore the need for improved communication and agreements between healthcare providers involved in opioid therapy. For instance, it is important to make agreements about who should inform the patient about risks of tolerance, dependency and addiction and how tasks are assigned in monitoring long-term opioid use and effectiveness.

#### 4.1.1 Strengths and limitations

The study included sufficient respondents to gain insight into the beliefs and experiences of PTs and the information provisions in community pharmacies during opioid counselling. In addition, we had a diverse sample PTs across the Netherlands. Based on the interviews, we identified several counselling activities that many PTs likely perform more often. This helps to highlight the activities that PTs may not be engaging in, which is evident in the survey results. One limitation is that participants were volunteers, so those with an interest in pain and opioid management may have been overrepresented. Recruitment via social media includes a risk of delusive response, to minimize this, participants were not compensated. Furthermore, the self-reporting nature of the questionnaire may have led to subjective and potential socially desirable responses.

#### 4.1.2 Practice implications

Pharmacy technicians require training to enhance their communication skills and knowledge about pain and pain treatment, including how to appropriately discuss risks like opioid dependence and tapering. Including the indication of opioids on prescriptions could support communication with the patient and promote safer use. By addressing existing barriers and leveraging facilitators including training for PTs and work protocols, the role of PTs in promoting safe opioid use and mitigating misuse risks can be optimized.

### 4.2 Innovation

While most studies focus on the role of physicians, pharmacists, and other healthcare providers in managing patients' opioid use, this research highlights a relatively underexplored group involved in opioid dispensing. In the Netherlands and many other countries, pharmacy technicians likely have the most regular face-to-face contact with patients who chronically use opioids. However, to our knowledge, there are no European studies investigating the role of PTs in opioid counselling. Understanding their role fills a critical gap and provides new insights into their potential for promoting appropriate opioid use. Highlighting barriers and facilitators related to PTs in opioid counselling is crucial for designing effective interventions that reach patients.

### 4.3 Conclusion

When dispensing opioids, pharmacy technicians typically counsel patients regarding dosage and common side effects. However, only around two-thirds of pharmacy technicians regularly address opioid dependency during first dispense, despite the importance they place on this topic. To close this gap and optimize the role of PTs in promoting safe opioid use, organizational and competency-related barriers must be addressed. This includes clear work protocols, communication training and including the indication on opioid prescriptions.

## Declaration of Generative Al and Al-assisted technologies in the writing process

During the preparation of this work the corresponding author used ChatGPT-4 in order to improve readability and English language. After using this tool, the authors reviewed and edited the content as needed and take full responsibility for the content of the publication.

## Funding statement

This work is part of the research project Tackling and Preventing The Opioid Epidemic (TAPTOE). This research received funding from the Dutch Research Council (NWO) in the framework of the NWA-ORC Call (NWA.1160.18.300). The views expressed in this paper are those of the authors and do not necessarily reflect the views or policies of the NWO. The NWO is not liable for any use that may be made of the information presented.

## Ethical approval

The research proposal was approved by the Utrecht University Institutional Review Board (division of Pharmacoepidemiology & Clinical Pharmacology) (UPF2201).

## CRediT authorship contribution statement

**Elsemiek A.W. Jansen-Groot Koerkamp:** Writing – review & editing, Writing – original draft, Visualization, Methodology, Formal analysis, Conceptualization. **Irem Simsek:** Writing – review & editing, Software, Investigation, Formal analysis, Data curation, Conceptualization. **Eman Badawy:** Writing – review & editing, Software, Formal analysis, Conceptualization. **Mette Heringa:** Writing – review & editing, Supervision, Resources, Project administration, Methodology, Conceptualization. **Marcel L. Bouvy:** Writing – review & editing, Supervision, Methodology, Conceptualization.

## Declaration of competing interest

None declared.

## Appendix A Supplementary data

Supplementary files Image 1
