# Supplementary material for: Patient counselling on opioids by pharmacy technicians: A mixed-method study to explore facilitators and barriers
Source: PEC Innov. 2025 Feb 17;6:100382. doi: 10.1016/j.pecinn.2025.100382 (PMC11904593; doi:10.1016/j.pecinn.2025.100382)
Supplement: Supplementary file 1 — Supplementary files [file mmc1.pdf]

Supplementary files

Supplementary file 1	Interview guide
Supplementary file 2	Questionnaire
Supplementary file 3	Table S1. Interviewed pharmacy technicians’ characteristics
Supplementary file 4	Tables S2, S3, S4, S5, S6, S7

Table S2. Provided information at first dispense of an opioid
Table S3. Provided information at first refill of an opioid
Table S4. Potential barriers to discuss the risk of opioid dependence in long-term opioid use
Table S5. Beliefs of pharmacy technicians on long-term use of opioids
Table S6. Associations between background characteristics and dispensing behavior (discussing risk of opioid dependence)
Table S7. Associations between beliefs of PTs about LTOT and dispensing behavior (discussing risk of opioid dependence)

Supplementary file 1 Interview guide

Introduction/aim of the interview:
I am *, a master's student/researcher in Pharmacy at Utrecht University. Over the years, the chronic use of opioids in the Netherlands has increased significantly, as you may already know. We are curious about the information you, as a pharmacy technician, provide to patients who collect opioids at the pharmacy. We are also interested in the considerations you make in doing so. For this research, we are interviewing multiple pharmacy technicians. The results will be used to develop a questionnaire that will be sent to more technicians. The interview will take 15 minutes. The interviews will be coded and processed. The interview will be recorded so it can be transcribed afterward. This recording will not be made available to others and will be destroyed after the study. Do you agree to this?

- Do you have any questions in advance?

Initial questions:
- What is your gender?
- What is your age?
- How long have you been working as a pharmacy technician in a community pharmacy?
- What type of pharmacy do you work in? (e.g., chain, independent, urban, rural, solo, health center)

Discussion with patients:
- What do you discuss during the first dispensing of opioids?
- Do you verify the indication with the patient during the first dispensing? Why or why not?
    - Do you also ask what kind of pain the patient has?
    - Do you ask what the doctor has already explained about it? If yes, what is usually communicated by the doctor to the patient?
    - How long do you think a patient should use opioids?
- What do you discuss during a second dispensing of opioids?
- Do you inquire about the current use and the use of other painkillers during the first and second dispensing?
- Do you mention the risks of chronic opioid use during the first and second dispensing? If yes, what do you explain?
- Where do you get the information you share during the first dispensing of opioids? (e.g., EU leaflet)
- On what basis do you provide information or ask questions during the second dispensing? (e.g., TU leaflet)
- Do you verify the indication with the patient during the second dispensing? Why or why not?
- Do you think the source of information for the first and second dispensing needs adjustment? If yes, what adjustments?
- What supporting materials are provided to the patient? (e.g., brochures, handouts)
- If you notice that the patient comes to collect their opioid medication earlier than expected (based on the system), would you discuss this with the patient, the pharmacist, or the doctor? Why or why not?
- If a doctor prescribes an initial opioid prescription for one month, do you dispense medication for one month or two weeks?

Considerations of pharmacy technicians:
- Are there topics you do not discuss with the patient but wish you could?
    - If yes, which topics?
- Are there topics related to the side effects of opioid use that you find difficult to discuss with the patient?
    - If yes, which topics and why?
- Are there other reasons why you discuss certain side effects with the patient and not others?
- Do you differentiate the information you provide to the patient about side effects depending on the indication (e.g., oncological patients/chronic pain)?
    - If yes, what distinctions do you make?

Opinions of pharmacy technicians:

- What are your views on the chronic use of opioids by patients?
- If you had to use an opioid chronically yourself, how would you feel about it?
    - Why?
- Would you like additional training on opioids? If yes, what would you like to learn?
- Would you like training on how to discuss opioids with patients? If yes, what would you like to learn?

End of interview:
Summarize the interview briefly and ask for any additional comments or suggestions that have not yet been covered during the interview.

Supplementary file 2 **Questionnaire**

Dear participant,

The number of patients who are using opioids chronically is increasing. They are often unaware of the negative effects of this. As a pharmacy technician, you regularly interact with these patients. Utrecht University and the SIR Institute for Pharmacy Practice and Policy are therefore conducting research into your experiences with counseling patients who collect opioids at the community pharmacy.

Completing this questionnaire will take approximately 10 minutes and is anonymous. We are seeking your opinion and experience; there are no right or wrong answers.

NB: In this questionnaire, the term opioids refers to strong opioids such as oxycodone, fentanyl, and morphine. Less potent agents such as tramadol and codeine are not considered here. In this questionnaire, long-term use of opioids is defined as continuous use for longer than three months.

I have read the above and agree to the processing of the responses for research
o Yes

Thank you kindly in advance for your cooperation on behalf of,
Irem Simsek (UU)
Dr. M. Heringa (SIR)
Prof. Dr. M.L. Bouvy (UU)
E. Badawy BSc (SIR)
E.A.W. Jansen Groot Koerkamp MSc (SIR/UU)

The following questions are about your work situation:

1) **Current position:**

o Pharmacy technician
o Pharmaceutical business administrator / pharmaceutical consultant
o Other, please specify:.........

2) **Age: ....**

3) **Gender:**

o Male
o Female
o Other

4) **How many years of work experience do you have in a public or outpatient pharmacy? ......**

5) **Is the pharmacy where you work located in a healthcare center?**

o Yes
o No
o Not applicable

6) **The pharmacy where you work is:**

o Chain pharmacy
o Independent pharmacy
o Outpatient pharmacy

7) In which province do you work?

o Groningen
o Friesland
o Drenthe
o Overijssel
o Flevoland
o Gelderland
o Utrecht
o Noord-Holland
o Zuid-Holland
o Zeeland
o Noord-Brabant
o Limburg

The following questions are about information provision during initial and subsequent dispensing consultations:

1) During a first dispensing consultation of opioids, I discuss the following:

		Never	Rarely	Sometimes	Often	Always
1	I ask about what the patient already knows about the opioid	1	2	3	4	5
2	Dosing regimen of the opioid	1	2	3	4	5
3	The duration of action of the opioid	1	2	3	4	5
4	How long the patient should use the opioid	1	2	3	4	5
5	The reason for prescribing (indication)	1	2	3	4	5
6	The possibility of experiencing constipation and the importance of using laxatives.	1	2	3	4	5
7	The possibility of experiencing nausea	1	2	3	4	5
8	Impact on reaction time	1	2	3	4	5
9	Advice to continue using other painkillers (such as paracetamol or NSAIDs)	1	2	3	4	5
10	Tolerance	1	2	3	4	5
11	Dependency	1	2	3	4	5

2) <u>During a second opioid dispensing consultation, I discuss the following:</u>

		Never	Rarely	Sometimes	Often	Always
1	The actual used dosage of the opioid	1	2	3	4	5
2	How long the patient should continue using the opioid	1	2	3	4	5
3	The reason for prescribing (indication)	1	2	3	4	5
4	The patient's experience with the effectiveness of the opioid	1	2	3	4	5
5	Dependency	1	2	3	4	5
6	The side effects experienced from the opioid	1	2	3	4	5

		Never	Rarely	Sometimes	Often	Always
3a	During the dispensing of opioids, I know whether the indication is cancer-related or non-cancer-related pain.	1	2	3	4	5
3b	I distinguish in my communication regarding opioids between patients experiencing cancer-related pain and those with non-cancer-related pain	1	2	3	4	5

<u>The following questions are about actions when there is a suspicion of inappropriate opioid use</u>

1) <u>I am concerned about opioid use when the patient:</u>

		Strongly disagree	Disagree	Neutral	agree	Strongly agree
1	has been using opioids for more than three months	1	2	3	4	5
2	returns before the end date for a repeat prescription of opioids	1	2	3	4	5
3	receives a higher dosage multiple times	1	2	3	4	5
4	visits the pharmacy regularly without a prescription	1	2	3	4	5

2) When I am concerned about opioid use, I take the following actions:

		Never	Rarely	Sometimes	Often	Always
1	I contact the prescriber	1	2	3	4	5
2	I consult with the pharmacist	1	2	3	4	5
3	I discuss opioid use with the patient	1	2	3	4	5
4	I take no action and dispense it	1	2	3	4	5
5	I make an agreement with the patient for dispensing times	1	2	3	4	5

The following questions are about your practical experience:

1) The number of patients in my pharmacy who I suspect may have dependence issues is:

- 0
- 1-5
- 6-10
- >10
- I don't know

2) The following considerations determine whether I discuss the risk of dependency with a patient regarding long-term opioid use:

		Strongly disagree	Disagree	Neutral	agree	Strongly agree
1	Feeling uncertain about discussing the risk of dependency with the patient	1	2	3	4	5
2	Insufficient knowledge to discuss all information	1	2	3	4	5
3	Fear of the patient's reaction	1	2	3	4	5
4	Not wanting to scare the patient	1	2	3	4	5
5	Not knowing exactly what the opioid is used for	1	2	3	4	5

6	No desire for information by some patients	1	2	3	4	5
7	Experience of limited time in the pharmacy	1	2	3	4	5
8	Belief that it is the responsibility of the physician to discuss this information with the patient	1	2	3	4	5
9	Insufficient privacy to discuss this sensitive information	1	2	3	4	5
10	Lack of pharmacy protocols regarding informing patient about opioids	1	2	3	4	5
11	Experience of a language barrier	1	2	3	4	5

Kindly specify your degree of agreement with the following statements regarding your vision on the long-term use of opioids

		Strongly disagree	Disagree	Neutral	agree	Strongly agree
1	I believe that patients are not adequately supported in the long-term use of opioids by the pharmacy team	1	2	3	4	5
2	I believe that the responsibility for long-term opioid use lies with the patient	1	2	3	4	5
3	I believe that opioids are prescribed and repeated by the physician too easily	1	2	3	4	5
4	It is important to discuss dependency as a standard risk of opioids with patients in the pharmacy	1	2	3	4	5

The following question is about your preferences for further education

I am in need of further education in the following areas (multiple answers possible)

- Knowledge about pain management and alternatives to opioids
- Knowledge about dependency and how to prevent it
- How to recognize patients with (risk of) addiction
- Communication training with motivational interviewing to encourage and support patients during opioid tapering
- I do not need further education
- Other, please specify.........

Do you have any comments?

....

End of survey

Supplementary file 3 Table S1

Table S1. Interviewed pharmacy technicians' characteristics

PT	age (in years)	years of experience	pharmacy location	area of pharmacy
1	29	7	solo	urban
2	55	34	solo	urban
3	50	30	health center	rural
4	64	40	health center	rural
5	29	10	health center	urban
6	38	18	solo	urban
7	36	14	solo	urban
8	36	15	health center	urban
9	50	30	health center	urban
10	46	0.7	health center	urban
11	24	4	solo	unknown
12	23	3	solo	urban
13	26	3	solo	urban
14	45	27	solo	rural
15	28	2	health center	rural
16	37	17	solo	urban
17	45	22	health center	rural
18	54	25	health center	urban

Supplementary file 4 Tables S2, S3, S4, S5, S6, S7

Table S2 Provided information at first dispense of an opioid (n=252) (% of respondents)

	never	rarely	sometimes	often	always
reason for prescription (indication)	9 (3.6%)	23 (9.1%)	89 (35.3%)	71 (28.2%)	60 (23.8%)
what the patient already knows about the opioid	15 (6.0%)	25 (9.9%)	74 (29.4%)	72 (28.6%)	66 (26.2%)
duration of opioid treatment	4 (1.6%)	29 (11.5%)	59 (23.4%)	83 (32.9%)	77 (30.6%)
risk of opioid dependence	(4.0%)	(9.9%)	(24.2%)	(31.0%)	(31.0%)
advice to (continue to) use other painkillers	9 (3.6%)	14 (5.6%)	57 (22.6%)	90 (35.7%)	82 (32.5%)
risk of opioid tolerance	7 (2.8%)	21 (8.3%)	49 (19.4%)	91 (36.1%)	84 (33.3%)
risk of nausea	10 (4.0%)	17 (6.7%)	53 (21.0%)	82 (32.5%)	90 (35.7%)
duration of action of opioid	3 (1.2%)	4 (1.6%)	22 (8.7%)	82 (32.5%)	141 (56.0%)
opioid dosing regimen	1 (0.4%)	2 (0.8%)	7 (2.8%)	31 (12.3%)	211 (83.7%)
risk of reduced ability to react	1 (0.4%)	0 (0.0%)	3 (1.2%)	19 (7.5%)	229 (90.9%)
constipation and relevance of laxantives	1 (0.4%)	0 (0.0%)	1 (0.4%)	17 (6.7%)	233 (92.5%)

Table S3: Provided information at first refill of an opioid (n=252) (% of respondents).

	never	rarely	sometimes	often	always
reason for prescription (indication)	29 (11.5%)	51 (20.2%)	98 (38.9%)	47 (18.7%)	27 (10.7%)
duration of opioid treatment	20 (7.9%)	37 (14.7%)	83 (32.9%)	69 (27.4%)	43 (17.1%)
risk of opioid dependence	21 (8.3%)	28 (11.1%)	93 (36.9%)	60 (23.8%)	50 (19.8%)
used dosage of opioid	7 (2.8%)	17 (6.7%)	60 (23.8%)	82 (32.5%)	86 (34.1%)
patients' experience with effect of opioid	5 (2.0%)	11 (4.4%)	49 (19.4%)	87 (34.5%)	100 (39.7%)
patients' experience with opioid side effects	6 (2.4%)	11 (4.4%)	45 (17.9%)	77 (30.6%)	113 (44.8%)

Table S4. Potential barriers to discussing the risk of opioid dependence in long-term opioid use (n=252) (% of respondents).

	strongly disagree	disagree	neutral	agree	strongly agree
insufficient privacy to discuss sensitive information	35 (13.9%)	99 (39.3%)	54 (21.4%)	53 (21.0%)	11 (4.4%)
experience of the language barrier	29 (11.5%)	69 (27.4%)	89 (35.3%)	57 (22.6%)	8 (3.2%)
insufficient knowledge to discuss all the information	33 (13.1%)	99 (39.3%)	52 (20.6%)	64 (25.4%)	4(1.6%)
experience of little time in the pharmacy	39 (15.5%)	87 (34.5%)	47 (18.7%)	58 (23.0%)	21 (8.3%)
physicians are responsible to discuss this information with the patient	30 (11.9%)	77 (30.6%)	61 (24.2%)	68 (27.0%)	16 (6.3%)
fear of patient's reaction	31 (12.3%)	80(31.7%)	56 (22.2%)	67 (26.6%)	18 (7.1%)
feeling of insecurity to discuss the risk of dependence	18 (7.1%)	57 (22.6%)	87 (34.5%)	84 (33.3%)	6 (2.4%)
lack of working agreements regarding information provision	36 (14.3%)	75 (29.8%)	50 (19.8%)	70 (27.8%)	21 (8.3%)
not wanting to scare the patient	14 (5.6%)	68 (27.0%)	71 (28.2%)	93 (36.9%)	6 (2.4%)
lack of information about indication	27 (10.7%)	56 (22.2%)	60 (23.8%)	97 (38.5%)	12 (4.8%)
patients not being open to receive information	11 (4.4%)	45 (17.9%)	72 (28.6%)	115 (45.6%)	9 (3.6%)

Table S5. Beliefs of pharmacy technicians on long-term use of opioids (n=252) (% of respondents)

statement	strongly disagree	disagree	neutral	agree	strongly agree
I believe that the patient is responsible for long-term opioid use	31 (12.3%)	138 (54.8%)	57 (22.6%)	23 (9.1%)	3 (1.2%)
I believe that patients are not guided sufficiently on long-term opioid use by the pharmacy team	9 (3.6%)	43 (17.1%)	60 (23.8%)	118 (46.8%)	22 (8.7%)
I believe that opioid are prescribed and repeated too easily by physicians	3 (1.2%)	13 (5.2%)	23 (9.1%)	109 (43.3%)	104 (41.3%)
It is important to discuss opioid dependence as a risk of opioids with patients in pharmacy as standard practice	2 (0.8%)	4 (1.6%)	14 (5.6%)	150 (59.5%)	82 (32.5%)

Table S6. Associations between background characteristics and dispensing behavior (discussing risk of opioid dependence)

Background characteristics		During first dispense PT discusses the risk of opioid dependence			
		never-rarely	sometimes	often-always	p-value
Gender	Female	33 (94.3%)	60 (98.4%)	154 (98.7%)	0.230
	Male	2 (5.7%)	1 (1.6%)	2 (1.3%)	
Job	Pharmacy technician	31 (88.6%)	57 (93.4%)	138 (88.5%)	0.529
	Pharmacy consultants	3 (8.6%)	3 (4.9%)	17 (10.9%)	
	Pharmacy manager	1 (2.9%)	1 (1.6%)	1 (0.6%))	
Age (years)	18-30	5 (14.3%)	8 (13.1%)	9 (5.8%)	0.194
	31-45	9 (25.7%)	11 (18.0%)	49 (31.4%)	
	45-60	18 (51.4%)	33 (54.1%)	73 (46.8%)	
	>60	3 (8.6%)	9 (14.8%)	25 (16.0%))	
Work experience (years)	0-10	9 (25.7%)	11 (18.0%)	14 (9.0%)	**0.025**
	11-20	6 (17.1%)	5 (8.2%)	33 (21.3%)	
	21-30	9 (25.7%)	16 (26.2%)	52 (33.5%)	
	>30	11 (31.4%)	29 (47.5%)	56 (36.1%)	
Location	In health center	20 (57.1%)	28 (45.9%)	76 (48.7%)	0.559
	Not in health center	15 (42.9%)	33 (54.1%)	80 (51.3%)	

Note: Statistical significance of differences tested by Chi-square test; Bold refers to p < 0.05 (significant).

Table S7. Associations between beliefs of PTs about LTOT and dispensing behavior (discussing risk of opioid dependence)

Attitudes of PTs		During first dispense PT discusses the risk of opioid dependence			
		never-rarely	sometimes	often-always	p-value
I believe that the patient is responsible for long-term opioid use	(strongly) disagree-neutral	28 (80.0%)	57 (93.4%)	141 (90.4%)	0.102
	(strongly)-agree	7 (20.0%)	4 (6.6%)	15 (9.6%)	
I believe that patients are not guided sufficiently on long-term opioid use by the pharmacy team	(strongly) disagree-neutral	13 (37.1%)	29 (47.5%)	70 (44.9%)	0.605
	(strongly)-agree	22 (62.9%)	32 (52.5%)	86 (55.1%)	
I believe that opioid are prescribed and repeated too easily by physicians	(strongly) disagree-neutral	6 (17.1%)	13 (21.3%)	20 (12.8%)	0.286
	(strongly)-agree	29 (82.9%)	48 (78.7%)	138 (87.2%)	
It is important to discuss opioid dependence as a risk of opioids with patients in pharmacy as standard practice	(strongly) disagree-neutral	5 (14.3%)	6 (9.8%)	9 (5.8%)	0.198
	(strongly)-agree	30 (85.7%)	55 (90.2%)	147 (94.2%)	

Note: Statistical significance of differences tested by Chi-square test; Bold refers to p < 0.05 (significant).
